# Paraprotein-Negative IL-1–Mediated Inflammatory Dermatosis: An Update on Schnitzler-Like Syndrome in the Absence of a Gammopathy

**DOI:** 10.1016/j.xjidi.2025.100405

**Published:** 2025-08-20

**Authors:** Rizwan Ahmad, Jean Dunne, Katie Ridge, Nicole Fagan, Niall Conlon

**Affiliations:** 1 UCARE Centre, Clinical and Diagnostic Immunology, St. James's Hospital, Dublin, Ireland; 2 School of Medicine, Trinity College Dublin, Dublin, Ireland

**Keywords:** Autoinflammatory disorders, Immunology, Medical dermatology, Schnitzler syndrome, Urticaria

## Abstract

Schnitzler syndrome (SchS) is a rare autoinflammatory condition characterized by chronic urticaria and systemic inflammation. Obligate diagnostic criteria include the presence of a monoclonal IgM or IgG band, with nearly all cases demonstrating a prompt response to IL-1 blockade. Recently, "Schnitzler-like" cases without a paraprotein have been reported. Although the exact nature of their relation to the original eponymous syndrome remains unclear, these cases share similar clinical features and response to IL-1 inhibition. Diagnostic delay is common in autoinflammatory syndromes, and the need to recognize potentially emerging cases is important. We present the case of a male aged 47 years with refractory urticaria, joint pain, and systemic inflammation resembling SchS but without detectable paraprotein, consistent with recently proposed paraprotein-negative IL-1–mediated inflammatory dermatosis (PANID). After failing conventional therapies, the patient achieved rapid and sustained remission with IL-1 blockade. This case underscores the importance of recognizing autoinflammatory syndromes, including PANID, in patients with refractory urticaria with associated inflammatory features. It also highlights the importance of a therapeutic trial of IL-1 inhibition.

## Introduction

Schnitzler syndrome (SchS) belongs to a growing category of systemic autoinflammatory disorders (SAIDs), arising from immune dysregulation. Despite its initial description in the 1970s, the precise genetic and molecular mechanisms underlying SchS remain poorly understood (Simon et al, 2013). Diagnosis is guided by the Strasbourg criteria, which require the presence of both a recurrent urticarial rash and a monoclonal IgM or IgG paraprotein for definitive classification (Simon et al, 2013). Minor criteria include recurrent fever, arthralgia or evidence of bone remodeling, neutrophilic dermal infiltrates on skin biopsy, and elevated serum inflammatory markers such as white cell count or C-reactive protein (CRP). A hallmark feature of SchS is the almost universal rapid response to IL-1 blockade, underscoring the pivotal role of IL-1 in its pathogenesis (Néel et al, 2014).

Diagnostic complexity has increased with the emergence of "Schnitzler-like" or "variant Schnitzler" syndromes, which lack the obligatory paraprotein but share clinical and inflammatory features of SchS. These cases also demonstrate a robust response to IL-1 inhibition (Bixio et al, 2021; Fagan et al, 2023; Zubiaga-Fernandez et al, 2024). The term paraprotein-negative IL-1–mediated inflammatory dermatosis (PANID) has been used recently to describe such presentations (Fagan et al, 2023).

Although there have been over 300 reported cases of SchS, with a recent rise due to increased awareness, there have been only 19 cases consistent with PANID reported to date (Table 1). PANID is likely underrepresented in literature owing to under diagnosis caused, in part, by its failure to align with established classification criteria.

**Table 1:** Cases of Schnitzler-Like Syndrome with Absent or Delayed Monoclonal Gammopathy

Age, y	Sex	Delayed Band	Hypergammaglobulinemia	Treatment with IL-1 Blockade	Response	Author
57	M	No	Polyclonal IgM and IgE	NA	NA	Husak et al (2000)
36	M	No	Polyclonal IgG	NA	NA	Varella et al (2005)
58	F	No	Polyclonal IgA and IgG	Anakinra	+	Treudler et al (2007)
54	F	No	Polyclonal IgA	Anakinra	+	Chu (2010)
44	F	No	None	Anakinra and canakinumab	+	Gladue et al (2014)
52	F	No	None	Anakinra	+	Zhou et al (2015)
64	M	No	Polyclonal IgA	Anakinra	+	Mensa-Vilaro et al (2016)
62	M	No	None	Anakinra	+	Urbanski et al (2016)
69	M	No	None	Anakinra	+	Ahn et al (2018)
21	F	No	Polyclonal IgM	Anakinra	+	Bixio et al (2021)
43	F	No	None	Canakinumab	+	Fujita et al (2021)
62	F	No	Polyclonal IgA	Anakinra	+	Fagan et al (2023)
75	M	No	None	Anakinra	+	Wesselmann et al (2023)
47	F	No	None	Anakinra	+	Ghali et al (2024)
47	M	No	None	Anakinra	+	This report
51	M	Yes	Delayed IgM after 4 years	Anakinra	+	Henning et al (2020)
58	M	Yes	Delayed IgM after 20 mo	Canakinumab	=^1^	Gladue et al (2014)
71	M	Yes	Delayed IgM κ after >2 years	Anakinra	+	Mulla and Neame (2015)
47	F	Yes	Delayed IgM κ after 5 years	Anakinra	+	Fagan et al (2023)

Abbreviations: F, female; M, male; NA, not available.

Presented is a summary of reported cases in the literature in chronological order. Cases with delayed monoclonal gammopathy are included at the bottom.

1 equates equivocal response.

## Pathophysiology

Although incompletely understood, SchS is thought to be driven by dysregulated activation of the innate immune system. As with some other autoinflammatory disorders, such as Cryopyrin-associated periodic fever syndromes (CAPS), activation of the NLRP3 inflammasome is thought to be central to the pathogenesis of SchS. NLRP3 pathway activation results in the elaboration of active forms of IL-1 and IL-18 with downstream amplification of the inflammatory cytokine response (Masson Regnault et al, 2020; Pizzirani et al, 2009). However, the inflammasome activation occurs without germline *NLRP3* sequence variation in most classical cases, distinguishing it from CAPS. IL-1 blockade with anakinra or canakinumab leads to rapid and near-complete symptom control, reinforcing IL-1 as the central inflammatory driver (Néel et al, 2014). With regard to IL-18, emerging evidence suggests that elevated levels may play a role in B-cell activation and survival, potentially linking the autoinflammatory and lymphoproliferative features of the disease (Airoldi et al, 2004; Kinoshita et al, 2006; Migliorini et al, 2009). Notably, despite effective suppression of symptoms with IL-1 inhibitors, the monoclonal gammopathy typically persists, indicating that the paraprotein is not simply a reactive epiphenomenon but a coexisting immunological process (Braud and Lipsker, 2024).

Although comparative studies examining the pathogenesis of SchS versus PANID remain limited, emerging case-based evidence hints at important distinctions. In SchS, a subset of patients has been found to carry the *MYD88* L265P sequence variant, linking the disease to B-cell neoplasms such as Waldenström’s macroglobulinemia and suggesting a potential shared origin between the inflammatory and lymphoid components (Pathak et al, 2019; Varettoni et al, 2013). Conversely, although reported in only a minority of cases, some patients with PANID have been found to harbor somatic mosaic sequence variations in the *NLRP3* gene, typically restricted to the myeloid lineage and resembling those seen in adult-onset CAPS (De Koning et al, 2015; Rowczenio et al, 2017; Zhou et al, 2015). These findings, together with some reports of patients who subsequently develop a monoclonal paraprotein over time (Table 1), support 2 potential interpretations of the nature of PANID: first, that it may represent a mild, acquired autoinflammatory phenotype in cases driven by somatic *NLRP3* sequence variations; and second, that it may serve as a precursor stage to SchS, in which the monoclonal gammopathy has not yet emerged at the time of diagnosis.

## Presentation

An appreciation of the presentation of autoinflammatory syndromes is vital given the breadth of medical specialties to whom these patients can present. Clinical symptoms such as fever, rash, and joint pain are nonspecific. However, timely diagnosis and initiation of appropriate treatment is essential because prolonged uncontrolled inflammation carries significant risks, including the development of AA amyloidosis, irreversible joint damage, and cardiovascular complications (Conlon and Edgar, 2014; Fagan et al, 2023). This underscores the need for increased awareness of such syndromes, especially in clinicians treating complex and refractory urticaria.

As an update to the small suite of published cases, we present a further case of a SchS-like syndrome without a paraprotein, consistent with PANID, exhibiting a swift response to IL-1 inhibition.

## Case Report

**Figure 1:**
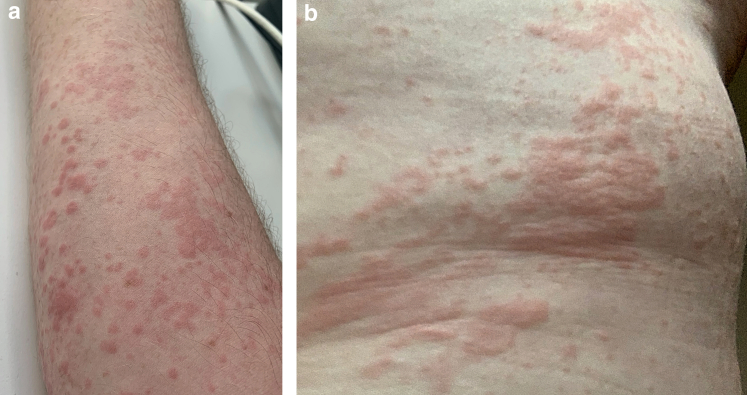
**Urticarial rash on the patient’s arm and torso.** (**a**) Patient’s arm. (**b**) Torso.

A male aged 47 years was referred to immunology services with a 2-year history of a persistent urticarial rash, which had not responded to antihistamines. Medical history included hypertension managed with 10 mg lercanidipine once daily. A prominent, pruritic, urticarial rash was distributed across the torso, lower back, and limbs (Figure 1). The patient reported recurrent joint pains affecting the wrists, fingers, and knees, which were associated with rash activity. There was no angioedema or temperatures.

Initial laboratory investigations revealed a normal white cell count (6.7 × 10^9^ l, normal range = 4.0–11.0 × 10^9^ l) and erythrocyte sedimentation rate (10 mm/hr, normal range = 0–15 mm/hr) with a mildly elevated CRP (7.37 mg/l, normal range < 5.00 mg/l). Immunoglobulin (Ig) levels were within normal limits (IgG = 11.3 g/l, IgA = 2.03 g/l, and IgM = 0.73 g/l) with no abnormal bands detected on serum protein electrophoresis. A range of blood tests, including antinuclear antibodies, serum-free light chains, total IgE, rheumatoid factor, anti-CCP, C3/C4, anti-C1q, and white cell differential, were normal.

The gentleman was commenced on high-dose antihistamines as per European Academy of Allergy and Clinical Immunology guidelines for management of chronic spontaneous urticaria (180 mg fexofenadine 4 times daily) (Zuberbier et al, 2022).

Clinical follow-up 1 month later revealed worsening rash and detrimental impact on QOL and sleep. The patient was commenced on 300 mg omalizumab at 4 weekly intervals. Repeat laboratory investigations were unchanged aside from an elevated CRP at 21.40 mg/l.

Over the next 10 months, the patient’s symptoms fluctuated. Poor response to omalizumab resulted in uptitration to 450 mg every 3 weeks. Urticaria control test scores were variable, but at no stage was effective control achieved (Zuberbier et al, 2022). Persistent rash and joint pains prompted reassessment. Differential diagnoses, including SAIDs or an urticarial vasculitis, were considered. This gentleman did not meet either Yamaguchi or Fautrel criteria for adult-onset Still’s disease (AOSD) (Fautrel et al, 2002; Yamaguchi et al, 1992).

Plain film x-ray of hands and knees revealed mild degenerative changes, with a small left-sided knee effusion. Skin biopsy showed mild dermal perivascular chronic inflammation without evidence of vasculitis. Immunofluorescence demonstrated patchy granular C3 deposition and was negative for IgG, IgA, IgM, or fibrinogen. Next-generation sequencing (NGS) for genes associated with autoinflammatory disorders, including CAPS, was negative.

A 48-hour trial of IL-1 inhibition therapy was initiated after confirming a negative IFN-gamma release assay (100 mg anakinra once daily). After 24 hours, the patient reported complete resolution of his rash and joint pain. CRP on the day of initiation was 33.84 mg/l. This fell to 20.49 mg/l in 24 hours and normalized to 3.66 mg/l in 72 hours. Omalizumab treatment was stopped. Aside from some initial minor injection site–related issues that resolved with education on technique, the patient has not experienced any side effects to date. At most recent follow-up, 1 year after the commencement of IL-1 blockade, the patient remains completely symptom free. His inflammatory markers are normal, and his Ig levels, electrophoresis findings, and serum-free light chains are within normal limits.

## Discussion

This case underscores the critical importance of recognizing autoinflammatory syndromes such as PANID in refractory urticaria with systemic inflammation. It remains pertinent to consider other SAIDs and connective tissue diseases in the differential, particularly AOSD, given the overlap in clinical presentation. In this case, key distinguishing features supporting PANID included an urticarial rash rather than AOSD’s classic ‘salmon-pink’ rash and the failure to meet AOSD criteria.

Whether PANID is a distinct clinical entity or represents a precursor to SchS remains uncertain. NGS remains a valuable tool in such cases to rule out the late presentation of hereditary autoinflammatory conditions. Uncertainties remain about optimum approaches for identification of genetic variants and somatic changes that might signal a predisposition to lymphoproliferative disorders or autoinflammation.

There are currently no guidelines on the frequency of monitoring Igs in these patients. Improved understanding may enable better prediction of who may go on to develop a monoclonal gammopathy, allowing more accurate discussions of prognosis. Early identification and timely initiation of IL-1 blockade could significantly alter disease course and improve patient outcomes, supporting the value of thorough evaluation in these challenging cases. The absence of a monoclonal gammopathy in the setting of refractory urticaria associated with autoinflammation should not preclude a therapeutic trial of anakinra.

## Ethics Statement

This submission was registered with the local Research & Innovation office (ID 5455). A full ethics submission is not required in our centre for case reports or series of <5 patients, but full informed written consent was obtained from the patient, including consent to the publication of images.

## Data Availability Statement

No datasets were generated or analyzed during this study. The authors affirm that all data necessary for confirming the conclusions of the article are present within the article, the figure, and the table.

## ORCIDs

Rizwan Ahmad: http://orcid.org/0000-0002-2032-0293

Jean Dunne: http://orcid.org/0000-0001-5816-3855

Katie Ridge: http://orcid.org/0000-0003-4276-7050

Nicole Fagan: http://orcid.org/0000-0001-7283-6171

Niall Conlon: http://orcid.org/0000-0002-1102-0758

## Conflict of Interest

The authors state no conflict of interest.
